# Involvement of FAM83 Family Proteins in the Development of Solid Tumors: An Update Review

**DOI:** 10.7150/jca.83420

**Published:** 2023-06-26

**Authors:** Yi Jiang, Jiahui Yu, Tong Zhu, Jiawen Bu, Yueting Hu, Yang Liu, Xudong Zhu, Xi Gu

**Affiliations:** 1 Department of Oncology, Shengjing Hospital of China Medical University, Shenyang, 110004 Liaoning province, P.R. China.; 2 Department of Ultrasound, Shengjing Hospital of China Medical University, Shenyang, Liaoning 110004, P.R. China.; 3 Department of General Surgery, Cancer Hospital of China Medical University, Cancer Hospital of Dalian University of Technology, Liaoning Cancer Hospital and Institute, Shenyang, Liaoning 110042, P.R. China.

**Keywords:** FAM83, tumor cells, abnormal expression, signaling pathway, therapeutic target

## Abstract

FAM83 family members are a group of proteins that have been implicated in various solid tumors. In this updated review, we mainly focus on the cellular localization, molecular composition, and biological function of FAM83 family proteins in solid tumors. We discussed the factors that regulate abnormal protein expression and alterations in the functional activities of solid tumor cells (including non-coding microRNAs and protein modifiers) and potential mechanisms of tumorigenesis (including the MAPK, WNT, and TGF-β signaling pathways). Further, we highlighted the application of FAM83 family proteins in the diagnoses and treatment of different cancers, such as breast, lung, liver, and ovarian cancers from two aspects: molecular marker diagnosis and tumor drug resistance. We described the overexpression of *FAM83* genes in various human malignant tumor cells and its relationship with tumor proliferation, migration, invasion, transformation, and drug resistance. Moreover, we explored the prospects and challenges of using tumor treatments based on the FAM83 proteins. Overall, we provide a theoretical basis for harnessing FAM83 family proteins as novel targets in cancer treatment. We believe that this review opens up open new directions for solid tumor treatment in clinical practice.

## Introduction

Cellular signaling pathways regulate cellular function through complex and ordered interactions 1. When one of these pathways is affected, the cells may become cancerous. Identification of key oncogenic signaling pathways is critical for identifying cancer therapeutic targets and improving survival 2, 3. Hence, the search for key signal proteins and their downstream signal factors is increasingly important. Although many of the key signaling pathways through which the tumor cells function and their corresponding targeted therapeutic drugs have now been discovered, efforts to identify new potential therapeutic targets should continue 4, 5. Owing to reports about FAM83 (a family with sequence similarity 83) proteins being potentially associated with the development of solid tumors, we introduced these proteins as therapeutic targets.

The FAM83 protein family consists of eight members (A-H), classified according to sequence similarity of conserved domains of unknown function, namely the DUF1669 domain 6-8. Determining the biochemical role of the DUF1669 domain is important for unraveling the biological role of the FAM83 protein family. The α, α-like, δ, and ε isoforms of casein kinase 1 (CK1) were found to interact with each member of FAM83 family proteins and played important roles in many aspects of intracellular homeostasis, including cell cycle progression, circadian rhythms, survival, DNA damage repair, membrane transport, and integration of signaling processes 9, 10. They were located in several locations in the cell, including the plasma membrane, cytoplasm, nucleus, actin cytoskeleton, and mitotic spindle. The increased catalytic activity of CK1 isoforms was associated with the development of solid cancer 11-14.

This review highlighted the cellular localization, molecular composition, and biological function of FAM83 family proteins in solid tumors. It discusses the factors that regulate aberrant protein expression and functional activity changes in solid tumor cells, including non-coding microRNAs and protein modifiers. Additionally, possible tumorigenesis mechanisms are examined, including the mitogen-activated protein kinase (MAPK) pathway, wingless/integrated (Wnt) pathway, and transforming growth factor beta signaling pathways. Moreover, the applications of FAM83 family proteins in the diagnosis and treatment of solid tumors were reviewed based on two aspects: molecular marker diagnosis and drug resistance. The overexpression of *FAM83* Gene in a variety of human malignant tumor cells and its relationship with tumor proliferation, migration, invasion, transformation, and drug resistance has also been described. In addition, this study explored the prospects and challenges of FAM83-based cancer therapy. It provided a theoretical basis for the use of FAM83 family proteins as novel targets for cancer therapy.

## Structures, intracellular proteins distribution, and basic biochemical and molecular properties of FAM83 family genes

**Figure 1:**
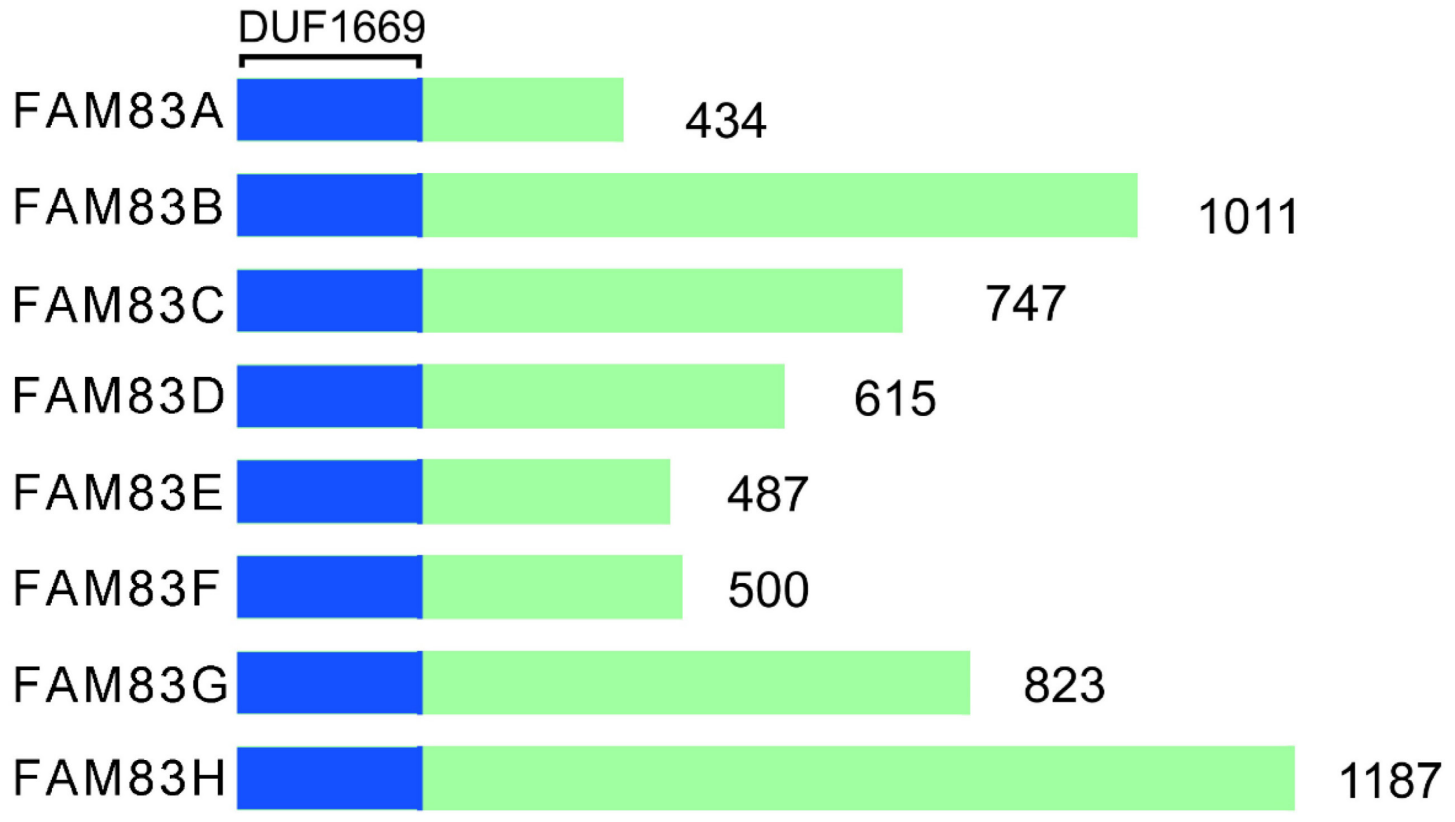
Structural representation of FAM83A-H.

The FAM83 (FAMily with sequence similarity 83) proteins consists of eight gene members, A-H. Each protein of FAM83 family of proteins mediates the interaction with the casein kinase 1 (CK1) subtype through the conserved domain 1669 (DUF1669) 15-20. This domain is shared by FAM83 family and affects many aspects of intracellular homeostasis, including cell cycle processes, circadian rhythms, survival, DNA damage repair, and the integration of membrane transport and signaling processes 10. The DUF1669 domain contains a phospholipase D (PLD)-like catalytic motif that is characterized by the presence of a His-x-Lys-x-Asp/Glu (also known as an HKD motif) where x denotes any amino acid 21. PLD can breakdown the membrane lipid phosphatidylcholine into choline and phosphatidic acid. The phospholipids are converted rapidly to diglycerides by phospholipase. Thereafter the protein kinase C subtype is activated 22. PLD is involved in phospholipid metabolism, intracellular signal transduction and vesicle transport 23-25. Typically, two such motifs are present in each PLD protein, with the two HKD motifs being clustered together to form the enzyme's catalytic core 26. FAM83 family has only one HKD motif; therefore, it has pseudo-PLD activity. In addition to FAM83D protein (also known as Chica) 27, the FAM83 protein lacks phospholipase catalytic activity. Only FAM83D protein is a true PLD enzyme with catalytic activity 28, 29. There are no similar sequences between family members outside the DUF1669 region, and each of FAM83 family member's C-terminal is composed of non-spherical disordered sequences. However, not all organisms have *FAM83* genes. For example, while some lower organisms, such as *Drosophila* 30, *Saccharomyces cerevisiae* and *Caenorhabditis elegans*, do not have *FAM83* genes, almost all of the *Vertebrata* with jaws encode *FAM83* genes 31. Furthermore, none of the FAM83 members share a similar sequence in the DUF1669 domain and the size differences are significant, ranging from 434 to 1,187 amino acids. Figure 1 shows the structural representation of FAM83 proteins, A-H.

With regard to the intracellular protein distribution in cancer cells, each FAM83 protein shows a different subcellular distribution pattern, including the plasma membrane, cytoplasm, nucleus, actin cytoskeleton and mitotic spindle, etc. FAM83A, also called BJ-TSA-9, is located mainly in the cytoplasm and perinuclear space; FAM83B, also called c60orf143, in the cytoplasm and plasma membrane; FAM83C protein, also called c20orf128, in the actin cytoskeleton; FAM83D protein, in the cytoplasm, nucleus, centrosome, and spindle; FAM83E protein, in the cytoplasm and perinuclear space; FAM83F protein, in the plasma membrane; FAM83G protein, also called PAWS1, in the cytoplasm and nucleus; and FAM83H protein, also called AI3, in the cytoplasm and cytoskeleton (**Table 1**).

Furthermore, from the GeneCards website (https://www.genecards.org) 32, 33, we preliminarily explored the available chromosomal loci of the gene family and their basic biochemical and molecular properties. *FAM83A* gene enabled identical protein binding activity, phosphatidylinositol 3-kinase regulatory subunit binding activity, and protein kinase binding activity, which are involved in cancer cell population proliferation and the epidermal growth factor receptor (EGFR) signaling pathway. *FAM83B*, enabled kinesin binding activity, microtubule binding activity, and protein kinase binding activity, which was involved in several processes, including positive regulation of cell cycle G1/S phase transition, protein localization to mitotic spindle, and regulation of intracellular signal transduction. *FAM83C* encoded a member of the protein family with sequence similarity 83 and is located at chr20:33,873,534-33,880,204. *FAM83D*, enabled EGFR binding activity, phosphatidylinositol 3-kinase binding activity, and protein kinase binding activity, which was involved in cell population proliferation and epidermal growth factor receptor signaling pathway, located in cytoplasm and membrane, and at chr6:54,711,569-54,806,820. *FAM83E* was located at chr19:48612880-48620644 (GRCh38/hg38) and may play a role in the MAPK signaling pathway. *FAM83F*, predicted to enable protein kinase binding activity, was located at chr22:40,390,953-40,439,533.

Similarly, *FAM83G,* predicted to have protein kinase binding activity, was involved in BMP signaling pathway, located in cytosol and nucleus, at chr17:18,872,102-18,908,117. Moreover, *FAM83H*, the protein encoded by this gene plays an important role in the structural development and calcification of tooth enamel, and defects in this gene were a cause of amelogenesis imperfecta type 3 (AI3). Besides, promoter DNA methylation may regulate the expression of FAM83A, FAM83D, FAM83F, and FAM83G mRNA in most CpG islands 34, 35.

## Associations between FAM83 family proteins and the development of solid tumors

### Lung cancer

The transcription levels of FAM83A/B/C/D/F/G/H genes are upregulated in non-small cell lung cancer (NSCLC) patients. The overexpression of FAM83A/B/D/F/H genes is associated significantly with the higher clinical stage of NSCLC. In addition, the high expression of FAM83A/B/C/D/F/H mRNA is associated significantly with shorter overall survival (OS) in patients with lung cancer, and the specificity/sensitivity of FAM83A, FAM83C, and FAM83H proteins in the diagnostic ability of death were found to be 0.9475/1,0.971897/1 and 0.9454545/0.8974359, respectively. In addition, a high mutation rate (51%) in the FAM83 protein was also observed in patients with lung adenocarcinoma (LUAD). Genetic alterations in FAM83 family of proteins were also found to be associated with shorter OS and disease-free survival (DFS) in LUAD patients 6. FAM83A was overexpressed in NSCLC, and its high expression was associated with a higher TNM stage and poor prognosis. Furthermore, its overexpression can promote proliferation, colony formation and invasion of NSCLC cells 36. The MYC and FAM83F proteins were two target genes of miR-1827 in the lung adenocarcinoma (LUAD) cells. The expression of these two genes was upregulated in the LUAD cells, and their overexpression was closely related to the progression of LUAD and the poor prognosis of patients 37. FAM83G was also up-regulated in NSCLC cells, promoting tumor cell proliferation 38.

With regard to the regulation mechanism, the overexpression of FAM83A activated the epithelial-mesenchymal transition (EMT) through the PI3K/AKT/snail pathway, and increased the expression of β-catenin and other Wnt pathway-related targeted genes. Furthermore, with the overexpression of FAM83A in the cells, the Hippo pathway activity was downregulated, while YAP1 and its downstream targets, cyclin E1 and the connective tissue growth factor (CTGF) were upregulated 39, 40. However, the use of Xav-939, an inhibitor of the Wnt signaling pathway, may reverse the stimulatory effects of FAM83A on YAP1, cyclin E1, and CTGF. By silencing the expression of FAM83A, opposite results may occur. Furthermore, when the expression of FAM83A is silenced, the use of the GSK3 inhibitor CHIR-99021 may restore the expression of YAP1, cyclin E1, and CTGF. These findings indicate that FAM83A may regulate the biological characteristics of NSCLC through the Wnt and Hippo pathways as well as their interaction.

**Figure 2:**
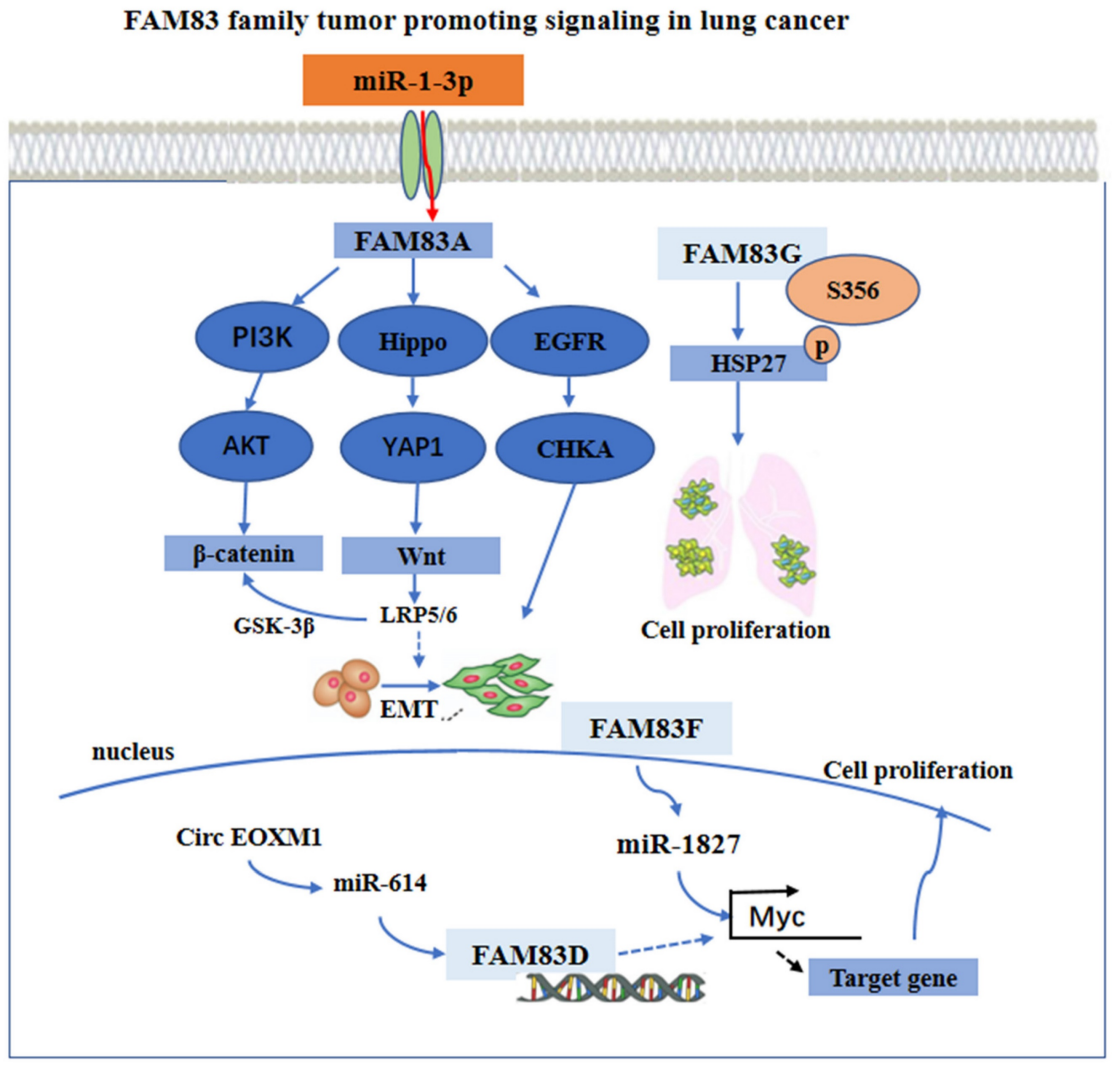
The functional roles and regulatory circuits of FAM83 family proteins in the progression of lung cancer. FAM83A promoted the development of lung cancer by PI3K/AKT, Hippo/YAP, and EGFR pathway. FAM83G induced phosphorylation of HSK to promote tumor cell proliferation. Regulation of FAM83D and FAM83F expression by ncRNA ultimately promoted cancer cell proliferation and other malignant behaviors.

Furthermore, FAM83A knockout may also inhibit the activation of an EGFR kinase/choline kinase α (CHKA) signal in the A549 and H1355 cells. The findings from using a bioinformatics method and, in addition, experimental validation, have indicated that the overexpression of FAM83A in lung cancer may be due to the down regulation of miR-1-3p. The miR-1-3p/ FAM83A axis may be modulated partially by the EGFR/choline phospholipid metabolic signaling pathway, thereby inhibiting the growth and motility of the lung cancer cells 41. Besides, FAM83D was a necessary gene in the circ FOXM1/miR-614 regulatory network. By interacting with miR-614, circ FOXM1 inactivated miR-614, further reducing the inhibition of FAM83D protein, thereby promoting the progression of NSCLC. The cirFOXM1/miR-614/FAM83D regulatory network may be a potential therapeutic target in patients with NSCLC 42. It has been found that miR-1827 was downregulated in LUAD, and that its low expression was closely related to the progression and poor prognosis of lung adenocarcinoma 43, 44. The overexpression of FAM83F proteins attenuated the effect of miR-1827 on the primary LUAD cells *in vitro*. In addition, miR-1827 inhibited tumor growth by decreasing the expression level of the MYC and FAM83F proteins *in vivo* 37. Besides, FAM83G acted as a counterpart of HSP27 and modulated HSP27 S356 phosphorylation to affect cell proliferation in NSCLC 38. These main regulatory mechanisms are summarized and shown in **Figure 2**.

### Gastric cancer

First, the expression level of FAM83B/C/D/H proteins in the gastric adenocarcinoma (GA) was upregulated significantly, while that of FAM83G/H proteins in GA was downregulated significantly. Second, the expression of FAM83C/D/G/H proteins has shown a significant correlation with the tumor stage in patients with GA. The mutation rate of FAM83H protein was found to be 46%, the highest in patients with GA among FAM83 family members. Lymph node metastasis is the most common mode of metastasis in the malignant progression of gastric cancer. FAM83C/D/G/H proteins play an important role in the prognosis of gastric cancer patients with lymph node metastasis. Among these patients with lymph node metastasis, FAM83C/D proteins with high expression were independent prognostic risk factors. The high expression of FAM83C/D proteins has also been closely related to the poor prognosis of patients with GA 7. As a result, the expression of FAM83B gene was upregulated, promoting the occurrence and metastasis of gastric cancer. In addition, the high expression of FAM83C gene was found to be associated significantly with shorter OS, DFS, and PPS in GA patients.

Furthermore, in the subgroup of patients with GA at different cancer stages, the high expression of FAM83C gene may also have a similar effect on the survival outcomes of patients with GA 7. In FAM83D protein, a microtubule-associated protein, the mRNA and protein levels were found to be upregulated in gastric cancer tissues with the cell lines showing a negative correlation with OS and DFS in patients with GA 45. In patients with GA, the expression of FAM83D protein showed a significant correlation with lymphocyte metastasis and the individual stage of the tumor 46. Furthermore, the overexpression of FAM83D protein in gastric cancer cells lines may increase the proliferation, invasion, and metastasis of gastric cancer cells *in vitro* and *in vivo*. A high expression of FAM83G mRNA was observed in gastric cancer; however, this was not significant. The level of expression of FAM83G protein was significantly lower in gastric cancer and was correlated with the individual cancer stage of patients with GA. In addition, the high expression of FAM83G proteins was associated with an improvement of OS and DFS in patients with GA 7. While FAM83H mRNA showed a significant overexpression in gastric cancer, its protein level was low, and was correlated significantly with the individual tumor stage in GA patients. Furthermore, high levels of FAM83H protein were associated significantly with better OS, PFS, and DFS in patients with GA 47.

**Figure 3:**
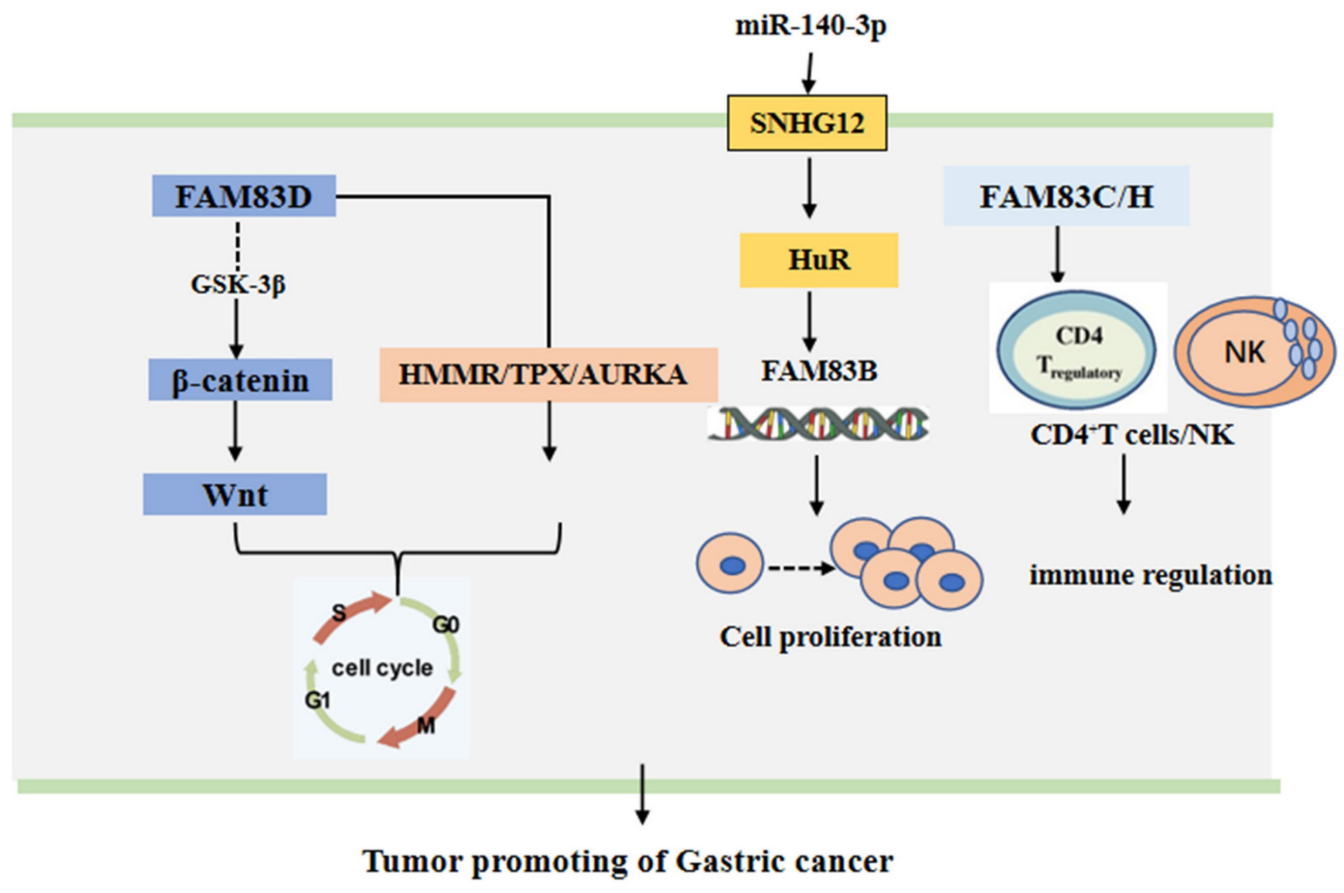
The functional roles and regulatory circuits of FAM83 family proteins in the progression of gastric cancer. FAM83D passed β-catenin/Wnt, HMMR/TPX, and other signaling pathways to regulate the cancer cell cycle; Under the regulation of small RNA, FAM83B can promote gastric cancer progression by regulating cell proliferation; FAM83C/H regulated CD4+ T cells and NK cells to affect anti-tumor immune response.

The FAM83 family of proteins has been shown to play a gastric cancer promoting role through different signal pathways. It has been found that miR-140-3p can bind directly to the small nucleolar RNA host gene 12 (SNHG12). This can down-regulate the expression of SNGH12, which can inhibit the translocation of HuR from the nucleus to the cytoplasm and increase the binding of HuR with the promoter of FAM83B gene 48. Moreover, the expression of FAM83D protein was cell cycle related. The knock down of FAM83D protein can arrest the cell cycle in the G0-G1 phase. Furthermore, FAM83D protein can bind with the hyaluronan mediated motility receptor (HMMR) 49, the targeting protein for Xklp2 (TPX2) 50 and Aurora kinase A (AURKA) 51, the drivers of mitosis. In addition, the knockdown of FAM83D protein was shown to be associated with the loss of activity of the Wnt/β-catenin signaling pathway 45. Additionally, FAM83H alone or in conjunction with SCRIB can stabilize β-catenin and stimulate the progression of GC 47. The CD4^+^ T cells and macrophages may be adverse prognostic factors of GA 52-55, while NK may be a favorable prognostic factor of GA 56, 57. However, when the expression of FAM83C protein was low, FAM83H protein, the CD4^+^ T cells and the macrophages have been found to have higher risk scores and the NK cells lower protective scores for the survival of patients with GA. This suggests that FAM83 family members may be involved in tumor immunity in GA 7. These main regulatory mechanisms are summarized and shown in **Figure 3**.

### Thyroid cancer

Although FAM83B has been previously considered an oncogene 58, its expression was found to be downregulated significantly in metastasized thyroid cancer (TC) tissues compared to normal and neoplastic thyroid tissues. The expression of FAM83B was related to thyroid cell differentiation and migration. Silencing FAM83B can increase the migration ability of the TC cells, while not affecting the RAS/MAPK/PI3K pathway which promoted the progression of the TC 59. FAM83F protein was a newfound protein that was highly expressed in parathyroid cancer (PTC). These researchers examined the expression of FAM83F protein in 106 cases of PTC, 34 cases of goiter and 41 cases of para-cancerous non-neoplastic thyroid tissues and found that 71% (76/106) of the PTC tissues showed overexpression of FAM83F protein in the cytoplasm, while the goiter tissue displayed overexpression of FAM83F protein in the nuclear tissue; however, FAM83F protein was not expressed in normal thyroid tissue 60. In this study, FAM83F protein was also identified as a new precancerous protein that was overexpressed in TC 60. In addition, compared with goiter, FAM83F protein in malignant thyroid tissue moved from the nucleus to the cytoplasm, leading to the formation of thyroid neoplasms.

TSH-induced goiter and *BRAF^T1799A^*-induced PTC animal models also showed an activation of FAM83F protein. After establishing a stable thyroid cell line PCCL3 with high expression of FAM83F protein, it was observed that FAM83F protein resulted in the thyroid follicular cells losing biological control, and the subsequent loss of the thyroid differentiation marker sodium iodide co-transporter and the reactivation of the stem cell markers, Lin28b and Sox2, which induced cell migration and resistance to the apoptosis induced by adriamycin. Additionally, FAM83F protein activated MAPK signaling pathways through interactions with BRAF and RAF, leading to the down regulation of the thyroid differentiation marker sodium iodide co-transporter 61, and inhibiting the transforming growth factor β (TGFβ) antiproliferative signal transduction in thyroid follicular cells. The TGFβ signaling pathway, which is generally unregulated in TC, is important in inhibiting anti-proliferative pathway associated with the EMT of invasive carcinoma, such as TC 62, 63. FAM83F regulated the biology and differentiation of the thyroid follicular cells through the cross-regulation of the MAPK and TGFβ signaling pathways. These indicated that the interactions between FAM83F protein and the cytoplasmic protein mediates the activation of different cancer-related signaling pathways.

FAM83F can interact with ELAVL1 (ELAV-like RNA-binding protein, also known as Hur). Hur is an RNA-binding protein that travels from the cytoplasm to the nucleus 64, 65. Its high expression has been associated with PTC malignant phenotypes 66, 67. Moreover, the expression patterns of FAM83F protein and Hur in thyroid neoplasms were similar. Although this suggested that the interaction between FAM83F and these pro-tumor proteins may play a role in the development of a thyroid neoplasm, further investigation was still required. In addition, the FAM83F mRNA 3'UTR contained multiple binding sites for miRNAs that were highly expressed in the normal thyroid, such as miR-143, miR125b, miR-29a, miR-30a, miR-30c, miR-30d and miR-22 (results from Encyclopedia of RNA Interactomes (ENCORI) database), These suggests that the down regulation of these miRNAs in TC may also increase the level of FAM83F protein, thereby promoting the development of TC. In summary, these results predicted that *FAM83* Genes were involved in TC progression through multiple pathways such as cell proliferation, apoptosis, and epigenetics and via a number of pro-tumor signal pathways.

### Breast cancer

In breast cancer, the expression of FAM83A, FAM83B, FAM83D, and FAM83G proteins was significantly higher than that in normal breast tissue, adversely affecting breast cancer patient's survival 8. These results provide preliminary evidence that FAM83 family gene may participate in the development of breast cancer. Notably, it has been found that there was a negative correlation between miR-613 and FAM83A expression. FAM83A can bind directly with miR-613 and participate in miR-613-mediated tumor suppression 68. In addition, the high expression of FAM83B was found to activate the MAPK signaling pathway, PI3K/AKT signaling pathway and the EGFR/PD-L1 axis in breast cancer 69, 70. Furthermore, the combined activation of the MAPK and PI3K/AKT pathways decreased the sensitivity of breast cancer cells to a variety of targeted therapies, including the EGFR, PI3K, Akt and mTOR inhibitors. Owing to the high expression of FAM83B resulted in intrinsic or selective drug resistance of tumor cells to genotoxicity or targeted therapy, the development of new therapies specific to FAM83 proteins may inhibit MAPK and PI3K/AKT signaling and the further expansion of drug-resistant tumor cells. Moreover, the high expression of FAM83B was found to be associated with the negative expression of ESR1 and PR. A negative ER/PR is a key attribute used to define more aggressive basal-like or triple-negative breast cancer subtypes that respond poorly to standard systemic chemotherapy 71, 72. Therefore, FAM83B high expression may affect the response to systemic chemotherapy of basal-like or triple-negative breast cancer. Besides, given that recent studies have shown that increased MAPK/ERK activity in basal-like breast cancer leads to increased proliferation even after neoadjuvant therapy 73, 74. Therefore, inhibiting key intermediates like FAM83B may enhance the sensitivity of tumor cells to respond to chemotherapy drugs.

**Figure 4:**
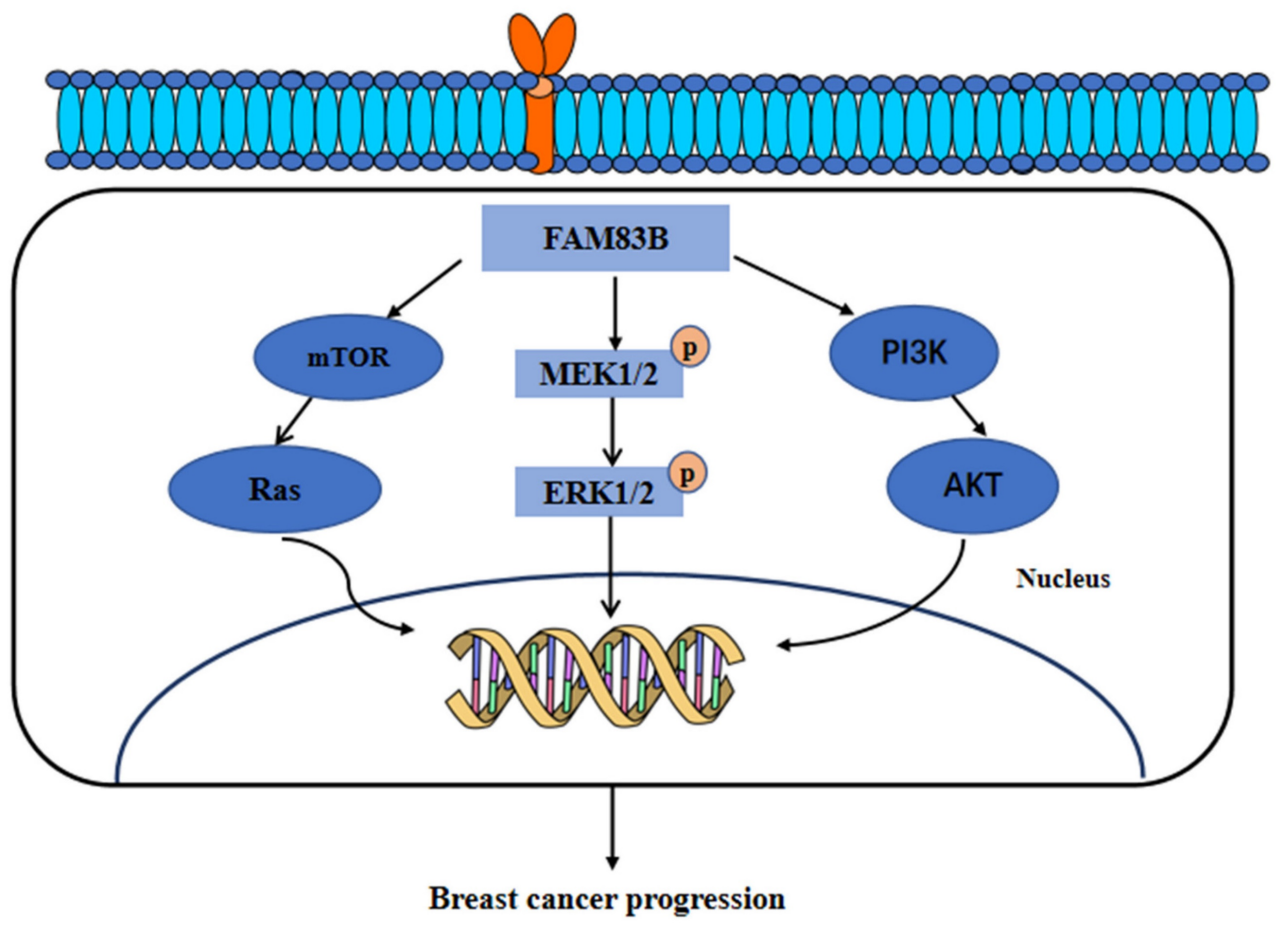
The functional roles and regulatory circuits of FAM83B in the progression of breast cancer. FAM83B can activate the mTOR/Ras, MEK/ERK, and PI3K/AKT signaling pathways to promote the malignant progression of breast cancer.

Furthermore, FAM83B have also been found to transform the normal human mammary epithelial cells into breast cancer cells through a RAS-dependent mechanism. FAM83B can bind with the RAS effector, CRAF, to activate the MAPK and mTOR signaling pathway. Moreover, the silencing of FAM83B may inhibit the proliferation and other malignant behaviors of breast cancer cells and the RAS-transformed human normal mammary epithelial cells. In addition, this study further identified that the high expression of FAM83B was associated with a higher tumor grade and poor survival outcomes 58. Additionally, our research recently identified the higher expression of FAM83A, FAM83D, FAM83F and FAM83G proteins in breast cancer tissues than in normal breast tissues. Furthermore, the genes co-expressed with FAM83A, FAM83D, FAM83F, and FAM83G proteins were mainly enriched in the Hippo, Hedgehog, and PI3K/AKT signaling pathways 8. In summary, these results suggested that FAM83 family proteins, especially FAM83B, can serve as therapeutic targets for breast cancer. These main regulatory mechanisms of FAM83B in the development of breast cancer are summarized and shown in **Figure 4**.

### Hepatocellular carcinoma

In hepatocellular carcinoma (HCC), the expression of FAM83A was upregulated. The high expression of FAM83A was found to be related to the poor prognosis of patients with HCC. FAM83A promoted the proliferation, invasion and migration *in vitro* and lung metastasis *in vivo* of the HCC cells 75. Additionally, the expression of FAM83D protein was also shown to be upregulated in patients with HCC^76^. These findings from a bioinformation analysis further indicated that FAM83D protein high expression was significantly associated with shorter OS and DFS in patients with HCC. The high expression of FAM83D protein was also more related to male patients and the advanced American Joint Committee on Cancer (AJCC) stage cases 77. The knockdown of FAM83D protein may inhibit the proliferation and invasion of the HCC cell lines Huh7 and HepG2 cells^76^. Moreover, Lin et al. found that FAM83D protein high expression was significantly related to a higher ratio of HCC recurrence after liver transplantation, a higher level of AFP and cancer stem cell marker expression 78. These findings indicated that FAM83A and FAM83D may act as driver genes, promoting the malignant behaviors of HCC.

**Figure 5:**
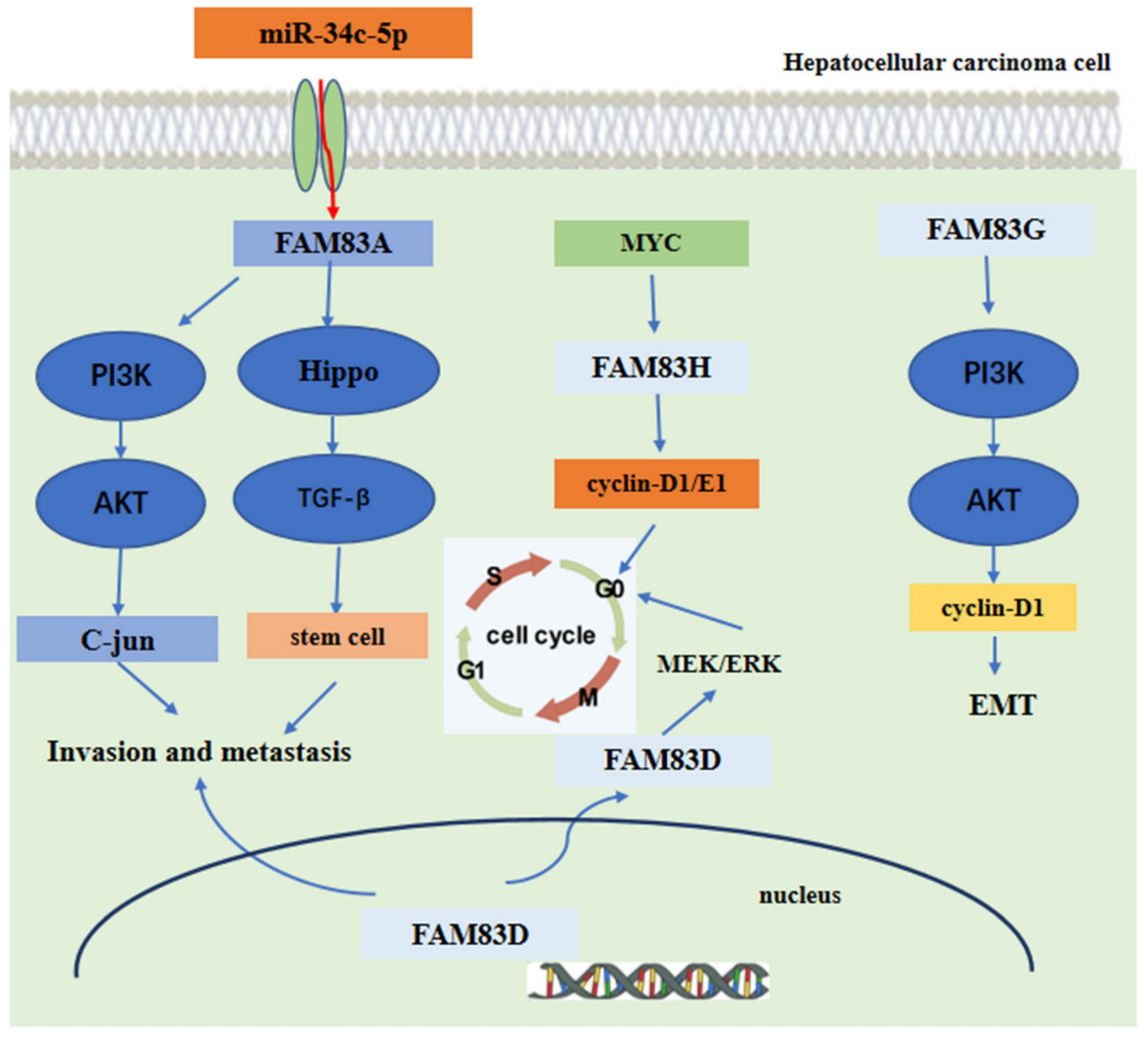
The functional roles and regulatory circuits of FAM83 family proteins in the progression of hepatocellular carcinoma. FAM83A/G regulated hepatocellular carcinoma cell invasion and metastasis via PI3K/AKT/c-JUN, Hippo/TGFβ/stem cell. FAM83H promoted liver cancer progression through CCND1/E1/MYC induced arrest of cell cycle. And FAM83D promotes hepatocellular carcinoma progression through MEK/ERK induced arrest of the cell cycle.

Mechanism analysis indicated that FAM83A could promote the expression of c-JUN by activating the PI3K/AKT/mTOR pathway. Moreover, the c-JUN protein directly binds to the promoter region of FAM83A gene, inducing FAM83 protein expression. Hence, these promotes a positive FAM83A/PI3K/AKT/c-JUN feedback loop to promote HCC invasion and metastasis 75. Furthermore, the expression of FAM83A was regulated directly by the tumor suppressor miR-34c-5p in HCC 79, 80. For FAM83D, the level of immune cell infiltration was shown to be related to FAM83D protein expression 81. Another research found that FAM83D protein can activate the MEK/ERK pathway to promote the cell cycle progression into the S phase, Finally, promoting the proliferation of HCC cells 82. Furthermore, FAM83D protein can also promote the expression of CD44 and CD44^+^ cancer stem cell malignancy through the MAPK, TGFβ, and Hippo signaling pathways 78. For FAM83G, the mechanism analysis showed that overexpression of FAM83G protein was associated with an over-activation of the PI3K/AKT pathway. FAM83G can activate the PI3K/AKT signal and promote PI3K phosphorylation by binding directly to the PI3K-P85 subunit, which increases the expression of cyclin D1 and decreases the expression of p21, at last, inducing the EMT process presented by the decreased expression of E-cadherin and increased expression of N-cadherin and snail 83. For FAM83H, the silencing of FAM83H protein may inhibit HCC cells proliferation, invasion and migration and may also decrease the expression of cyclin D1, cyclin E1, snail and MMP2. Furthermore, the expression of FAM83H protein was dependent on *MYC*, an oncogene 84-86, which can induce the transcription of *FAM83H* gene 84, 87-89. These main regulatory mechanisms of FAM83 family proteins in the development of HCC are summarized and shown in **Figure 5**.

### Esophageal cancer

A previous study has reported an increase in the level of FAM83F mRNA in esophageal squamous-cell carcinoma (ESCC) 90. Furthermore, miR-143 was found to be correlated negatively with the level of FAM83F mRNA in ESCC. miR-143 can bind directly to the 3'-UTR of FAM83F mRNA and regulate the expression of FAM83F protein. Furthermore, the high expression of miR-143 can inhibit the proliferation, migration and invasion of the ESCC cells by negatively regulating the expression of FAM83F protein, and inducing the G1/G0 arrest of the ESCC cells 90. Also, miR-455-3p was found to bind with the 3'-UTR of FAM83F mRNA and can promote the proliferation and migration of the ESCC cells by down-regulating the expression of FAM83F protein 91. Therefore, further investigation into the role of FAM83F protein in ESCC is recommended for future studies.

### Pancreatic ductal adenocarcinoma

The expression of FAM83A, FAM83B, FAM83D, FAM83E, and FAM83H proteins showed a significant increase in pancreatic ductal adenocarcinoma (PDAC) 92. Moreover, their increased expression was associated with late tumor stage or poor prognosis 93-95, suggesting that FAM83 genes may serve as oncogenes in PDAC. Furthermore, these FAM83 proteins exert their functions through different molecular mechanisms in PDAC. FAM83A can regulate the growth and death of the PDAC cells through MEK/ERK pathway. FAM83A/MEK/ERK pathway in PDAC cells can maintain its malignant behaviors 96. Additionally, the miR454-FAM83A-TSPAN1 axis promoted autophagy flux and mediated cooperation between WNT-CTNNB1 signaling and autophagy 97. The silencing of FAM83B in PDAC cells can arrest the cell cycle in the G0-G1 phase and inhibit cell proliferation. The silencing of FAM83B can also decrease tumorigenicity in nude mice 94. FAM83D protein knock down can inhibit the proliferation, mitochondrial respiration capacity, and aerobic glycolysis of the Wnt/β-catenin pathway in PDAC cells 93. A function enrichment analysis also showed that FAM83E and FAM83H proteins were involved in RAS GTP binding, Ras GTP activity regulation and RAS protein signal transduction. As an important member of the Ras family, KRAS is a key driver of PDAC, with an approximately 100% mutation rate 98, 99. SMAD4, another PDAC driving gene 100, 101, was found to be correlated significantly with the overexpression of FAM83E and FAM83H proteins. Moreover, the overexpression of FAM83E and FAM83H proteins was associated significantly with KRAS activation and SMAD4 deletion. In summary, FAM83E and FAM83H proteins may regulate KRAS or SMAD4 in PDAC, leading to cancer progression of PDAC 102. Besides, these authors also found that FAM83H may stimulate the Ras-PI3K-Akt-mTOR signaling pathway, promoting the development of PDAC 102.

Furthermore, FAM83 family proteins were not only biomarkers for predicting prognosis, but also reflected the anti-tumor immune status of PDAC in the tumor microenvironment. The expression of FAM83A, FAM83D, FAM83E, and FAM83H proteins has shown a significantly negative relationship with the infiltration of the CD8^+^ T cells and Gamma Delta T cells in PDAC. The expression of FAM83H protein was correlated with the level of tumor-infiltrating lymphocytes (TILs). Overexpression of FAM83H inhibited the infiltration level and anti-tumor activity of TILs, especially on CD8+ T cells. Furthermore, FAM83H overexpression was significantly associated with low expression of TILs related biomarkers such as CD8A, CD8B, CD2, CD3D, and CD3E 103-106. Additionally, it influenced immunomodulators (such as the effector CD4+ T cells, eosinophil granulocyte cells, mast cells, and follicular helper T cells), immunosuppressors (such as PVRL2, LGALS9, and IL10RB), immunostimulants (such as CXCL12, ENTPD1, CD28, and KLRK1), and MHC molecules (such as HLA-DPADOA, HLADPB1, HLA-DRA, and DRA). These results were mainly obtained from the TISIDB database 92, 107, 108. In conclusion, the FAM83 members are of great value in predicting the prognosis of PDAC, and they may play an important role in regulating tumor progression and the anti-tumor immune response of PDAC.

### Cervical cancer

Although the expression of FAM83A in cervical cancer was found to be higher than that in normal tissues 109, 110, its expression decreased in patients with advanced FIGO stage, deep stromal invasion, poor differentiation and/or lymph node metastasis. The expression of FAM83A negatively correlated with the survival time of patients with cervical cancer. *FAM83A* gene knockout can promote the proliferation, migration and invasion of Caski and HeLa cells. The Mouse Xenotransplantation model showed that *FAM83A* gene knockout promoted tumor growth *in vivo* 110. However, Liu et al. found that overexpression of FAM83A promoted the proliferation, invasion and migration of cervical cancer cells. FAM83A may serve as a therapeutic target for cervical cancer 109. Hence, the function of FAM83A in cervical cancer remains controversial. In addition, Chen et al. also discovered overexpression of FAM83H protein in cervical cancer, which was associated with poor survival outcomes 111.

In terms of molecular mechanism, RNA sequencing showed that a knockout of FAM83A increased the expression of integrin α-1, α-3, α-5, β-4 and β-5 both *in vivo* and *in vitro* in cervical carcinoma 110. Integrins are members of the glycoprotein family that form the ECM molecular heterodimer receptor. They were essential for cancer cell migration, invasion, and tumor metastasis, including cervical cancer 112-115. Therefore, FAM83A can affect the invasion and metastasis of cervical cancer by interacting with integrin α-1, α-3, α-5, β-4 and β-5. In addition, FAM83A promoted the activation of β-catenin, up-regulated the expression of the Wnt target genes *CD44*, *MYC*, *CCND1*, *MMP7*, and inhibited the expression and activation of GSK3β. It also promoted the EMT of cervical cancer cells, thereby enhancing their proliferation and invasion of cervical cancer cells 116. Besides, the FAM83H protein can also promote cervical cancer cell proliferation, invasion and migration through the PI3K/AKT pathway 111. In summary, both FAM83A and FAM83H contributed to the progression of cervical cancer and may serve as potential therapeutic targets.

### Ovarian cancer

The findings of a bioinformation analysis indicated that the expression of FAM83A/D/E/F/H mRNA was significantly upregulated in ovarian cancer. The high expression of FAM83D/H mRNA was also associated significantly with poor OS and progression-free survival 117. The results of immunohistochemistry analysis also indicated that the expression of FAM83H protein was related to the FIGO stage and the pathological subtype of ovarian cancer 117. Moreover, Zhao and et al found that the expression of FAM83A was upregulated in ovarian cancer and associated with poor survival outcomes 118. Conversely, low expression of FAM83B was linked to the poor survival of patients with ovarian cancer 119. For FAM83D, the expression of FAM83D protein was upregulated in ovarian cancer and associated significantly with poor survival outcomes in patients with ovarian cancer 120, 121. *In vitro* experiments also indicated that high expression of FAM83D protein significantly promoted proliferation, migration and sphere genesis of ovarian cancer cells 120. In summary, FAM83A/B/D may function as oncogenes in ovarian cancer.

**Figure 6:**
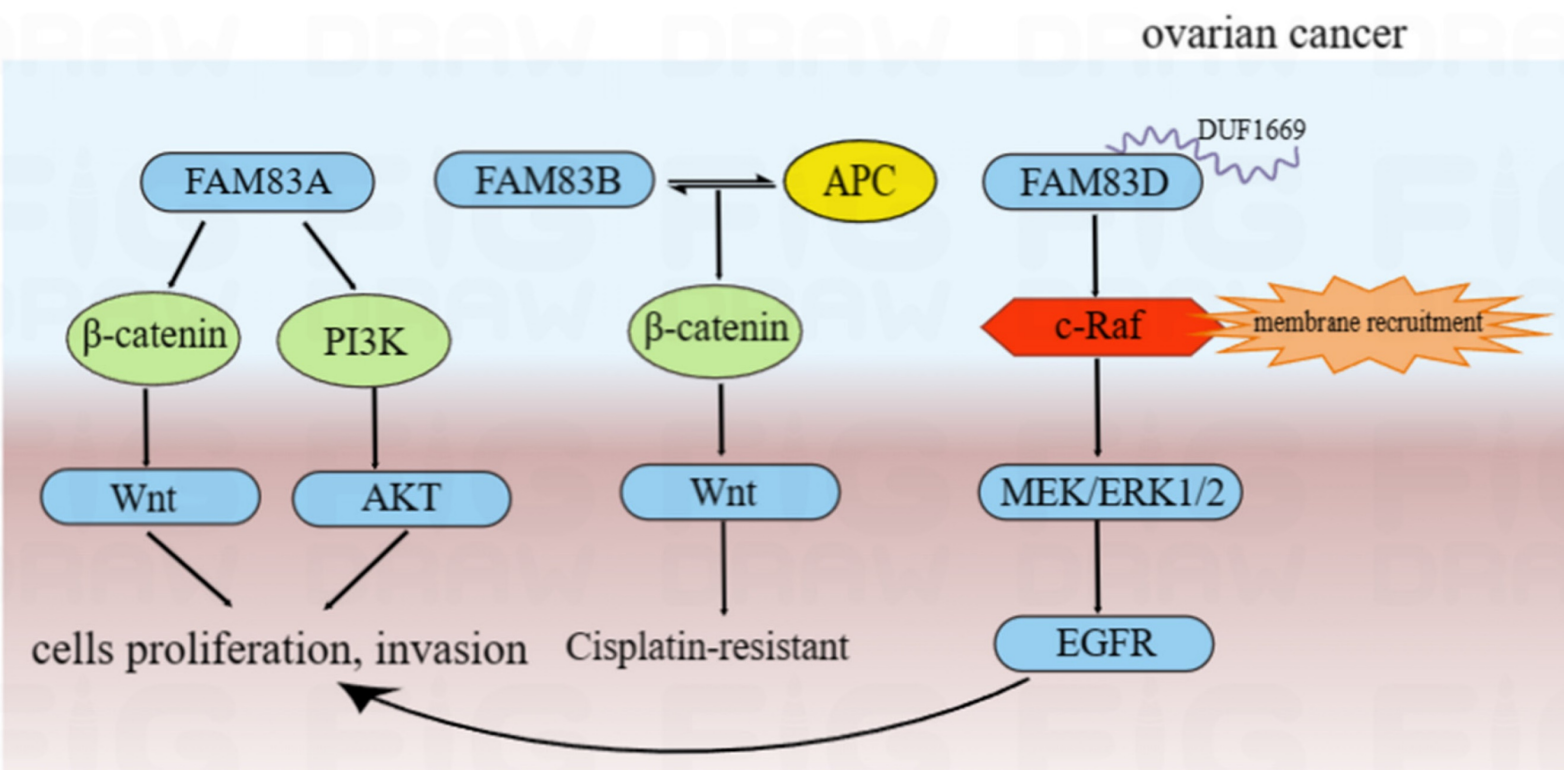
The functional roles and regulatory circuits of FAM83 family proteins in the progression of ovarian cancer. FAM83A can promote ovarian cancer cells proliferation and invasion by Akt/Wnt/β-catenin pathway; FAM83B may interact with APC to inhibit the Wnt pathway, thereby resulting in cisplatin resistance in the treatment of ovarian cancer; FAM83D can increase membrane recruitment in c-Raf and further activate the MEK/ERK1/2/EGFR pathway to promote proliferation and invasion of ovarian cancer cells.

The silencing of FAM83A protein may inhibit ovarian cancer cells proliferation, invasion and chemosensitivity through the Akt/Wnt/β-catenin pathway, while the overexpression of FAM83A protein had the opposite effect 118. FAM83B may interact with APC to inhibit the Wnt pathway, resulting in cisplatin resistance 119. Moreover, compared to routine ovarian cancer cell lines, the overexpression of FAM83D protein in the highly metastatic (NM) cells was validated and showed a stronger EGFR and c-Raf phosphorylation. This suggested that FAM83D protein can activate the EGFR pathway. We also found the more detailed action mechanisms of this process. The DUF1669 domain of the FAM83 protein was involved in the inhibiting of the retention of c-Raf. Thus, FAM83D protein can increase membrane recruitment in c-Raf and further activate the MEK/ERK pathway. MEK/ERK1/2 was activated by FAM83D protein and can up-regulate the expression of MMP2 and activate EGFR through a ligand-dependent mechanism, thereby leading to the invasion and metastasis of ovarian cancer cells 120. Furthermore, FAM83D protein can also inhibit autophagy and promote the proliferation and invasion of ovarian cancer cells via the PI3K/AKT/mTOR pathway and may be regulated directly by the potential tumor suppressor, miR-142-3p 121-123. From the above results, we can infer that FAM83 family proteins are involved in the development of ovarian cancer by regulating cell proliferation/cell cycle/autophagy via various tumor-related signal pathways. These main regulation mechanisms of FAM83 family proteins in the development of ovarian cancer were summarized and shown in **Figure 6**.

## Application of FAM83 family proteins in solid tumor diagnosis and treatment

The evaluation of human tumor samples has indicated that most of FAM83 family members were overexpressed in cancer, and that their gene expression was associated with specific cancer subtypes, suggesting that FAM83 may serve as novel biomarkers for solid cancer diagnosis and potential therapeutic targets for related solid tumors.

FAM83A was highly expressed in lung cancer, HCC, breast cancer, pancreatic cancer, bladder cancer, and ovarian cancer cells compared with normal tissues. And its expression level was significantly correlated with the survival and distant metastasis rates of patients with solid cancer 41, 82, 124. Besides, targeting the tumor specific effector protein FAM83A in RAS signal transduction can effectively inhibit the progression of PDAC 125. Similarly, FAM83B was highly expressed in breast cancer, bladder cancer, testicular cancer, ovarian cancer, thyroid cancer, and lung cancers 119, 126-128. Also, FAM83B high expression was linked to poor DFS in individuals with lung cancer 41. Quantitative examination of FAM83A and FAM83B mRNA levels using qPCR in 362 patients with NSCLC 36, revealed their high expression in NSCLC, suggesting their potential as predictive biomarkers for the diagnosis of NSCLC. High expression of FAM83C was found in lung cancer and GC. Besides, FAM83C high expression was significantly associated with a shorter OS of patients with GC 6, 7. High expression of FAM83D protein has shown high diagnostic values of breast and HCC 77, 129. The overexpression of FAM83D protein in GC cells also enhanced cell inclusion, circulation, migration and invasion. FAM83D protein was an effective biomarker for the development of GC. MYC-induced overexpression of FAM83F protein in LUAD has also been linked to its diagnostics and poor prognosis in patients with LUAD 37. For FAM83G, Kim et al. reported that its overexpression in HCC cells, and that its high expression predicted poor survival outcomes among patients with HCC. It can maintain the expression of cyclins D1 and E1, and suppress the conversion of p53, which affects the invasion and proliferation of HCC cells. Therefore, FAM83G protein may be a poor predictive marker for HCC 83. FAM83H expression was significantly upregulated in PDAC and positively correlated with higher histological grade, tumor recurrence, and poor prognosis of patients with PDAC 103. These results suggested that high expression of FAM83 family proteins may serve as diagnostic biomarkers, and targeting them could be a potential treatment strategy for the above solid tumors. The main diagnostic values of the FAM83 family proteins in solid tumors were summarized and presented in **Table 2**.

Drug resistance was a critical factor contributing to tumor morbidity and mortality, especially in solid tumors 130, 131. Various classes of drugs existed, such as cytotoxic drugs, immunomodulators, and hormonal drugs 132-138. FAM83 family proteins played important roles in the process of drug resistance and tumor treatment.

(1) In addition to acting as a biomarker, FAM83A protein can also affect the therapeutic effects of EGFR-tyrosine kinase inhibitors (EGFR-TKIs) in breast cancer 139. FAM83A can interact with and cause phosphorylation of c-RAF and PI3K p85, which are upstream of MAPK and downstream of EGFR, affecting the action of EGFR-TKIs. In mice inoculated with tumor cells overexpressing EGFR and treated with EGFR inhibitors, the endogenous FAM83A protein level and its DNA copy number significantly increased in surviving tumor cells, leading to resistance. In addition to its role in TKI resistance, FAM83A protein confers resistance to trastuzumab 140. Trastuzumab is a monoclonal antibody that was used to treat HER2 positive breast cancer 141, 142. After interference with FAM83A protein, these HER2 positive breast cancer cells were re-sensitive to trastuzumab 143. Therefore, targeted FAM83A could increase the sensitivity of breast cancer cells to EGFR-TKIs or trastuzumab.

(2) Similar to FAM83A, FAM83B also accelerated abnormal signal transduction and resistance to EGFR-TKIs in NSCLC 36. Besides, elevated expression of FAM83B activated PI3K/AKT/mTOR pathway and conferred a decreased sensitivity to PI3K, AKT, and mTOR inhibitors in solid cancer 144-146. Therefore, targeted FAM83A could increase the sensitivity of breast cancer cells towards EGFR-TKIs or PI3K, AKT, and mTOR inhibitors.

(3) Fuziwara et al. reported that papillary thyroid cancer cells with high expression of FAM83F protein exhibited increased resistance to Adriamycin-induced cytotoxicity and had fewer apoptotic cancer cells than control cells 60. This result suggests that targeting FAM83F in papillary thyroid cancer cells could increase the response to Adriamycin-induced cytotoxicity chemotherapy. In summary, targeting specific FAM83 family proteins, particularly FAM83A, FAM83B, and FAM83F, may positively affect solid tumor-related therapies by increasing the sensitivity to relevant drugs. This offers new hope for the treatment of solid tumors in clinical practice. The confirmed therapeutical values of the FAM83 family proteins in solid tumors are summarized and showed in **Table 3**.

## Conclusions and Outlook

Although the DUF1669 domain of each FAM83 member and its C-terminal sequence, as well as their specific functions have not yet been determined, detailed bioinformatics evaluation and experimental verification using C-terminal exchange deletion mutants of FAM83 provided crucial insights into these family proteins. Moreover, while the reasons underlying the upregulation of the FAM83 proteins in tumor cells remained obscure, a global evaluation has revealed that each of the FAM83 proteins played a distinct role in cancer-related signaling. However, it is recommended that, future research should focus on the unique function of each of the FAM83 proteins and develop novel strategies to enable specific targeted research and treatment. Most human proteome analyses have demonstrated that the FAM83 members exhibited elevated levels of expression in the body compared to other proto-oncogenes, such as EGFR and Ras. To date, published results have predominantly used the overexpression and RNAi methods in non-transformed and cancer cell lines. Currently, there is only one type of C57BL/6 mouse with a FAM83H gene knockout; however, this genotype is lethal two weeks after birth 147. Future research should develop knockout or transgenic models of other FAM83 proteins. Such animal models may provide practical genetic evidence for the normal development and homeostasis of each FAM83 family member. FAM83 proteins are potential biomarkers for some cancers, and the expression of the FAM83 members may provide a reference for therapeutic strategies and predict higher survival rates in patients with cancer. It is recommended that future research delineate the specific function of each of the FAM83 proteins and develop novel strategies to treat their unique carcinogenicity. This will provide new insights and open new paths for further clinical treatment of solid tumors.

## Figures and Tables

**Table 1:** The synonyms and subcellular localization of the FAM83 family proteins in cancer cells.

FAM83 family proteins	FAM83A	FAM83B	FAM83C	FAM83D	FAM83E	FAM83F	FAM83G	FAM83H
Synonyms	BJ-TSA-9	c60orf143	c20orf128	CHICA	/	/	PAWS1	AI3
Subcellular localization	Cytoplasm, perinuclear space	Cytoplasm, plasma membrane	Actin cytoskeleton	Cytoplasm, nucleus, centrosome, spindle	Cytoplasm, perinuclear space	Plasma membrane	Cytoplasm, nucleus	Cytoplasm, cytoskeleton

**Table 2:** The main diagnostic values of the FAM83 family proteins in solid tumors.

FAM83 family proteins	Main diagnostic values in solid tumors
FAM83A	Highly expressed in lung cancer, HCC, breast cancer, pancreatic cancer, bladder cancer, and ovarian cancer. It can be effective marker of tumor progression and adverse clinical prognosis.
FAM83B	Highly expressed in breast cancer, bladder cancer, testicular cancer, ovarian cancer, thyroid cancer, and lung cancer. It can be effective marker of tumor progression and adverse clinical prognosis.
FAM83C	Highly expressed in lung cancer and GC. It can be effective marker of tumor progression and adverse clinical prognosis.
FAM83D	Highly expressed in breast cancer, GC and HCC. It can be effective marker of tumor progression.
FAM83E	Highly expressed in PDAC.
FAM83F	Highly expressed in LUAD. It can be effective marker of tumor progression and adverse clinical prognosis of patients with LUAD.
FAM83G	Highly expressed in HCC. It can be effective marker of tumor progression and adverse clinical prognosis of patients with HCC.
FAM83H	Highly expressed in PDAC and ovarian cancer. It can be effective marker of tumor progression and adverse clinical prognosis of patients with PDAC.

**Table 3:** The confirmed therapeutical values of the FAM83 family proteins in solid tumors.

FAM83 family proteins	Confirmed therapeutical values in solid tumors
FAM83A	Targeted FAM83A could increase the sensitiveness of breast cancer cells towards EGFR-TKIs or trastuzumab.
FAM83B	Targeted FAM83A could increase the sensitiveness of breast cancer cells towards EGFR-TKIs or PI3K, AKT, and mTOR inhibitors.
FAM83C	/
FAM83D	/
FAM83E	/
FAM83F	Targeted FAM83F in papillary thyroid cancer cells could increase the respond to Adriamycin-induced cytotoxicity chemotherapy.
FAM83G	/
FAM83H	/
